# A sheet pocket to prevent cross-contamination of formalin-fixed paraffin-embedded block for application in next generation sequencing

**DOI:** 10.1371/journal.pone.0266947

**Published:** 2022-05-04

**Authors:** Keiichi Iwaya, Hisae Arai, Nanao Takatou, Yuka Morita, Rinko Ozeki, Hirofumi Nakaoka, Masaru Sakamoto, Tsutomu Kouno, Masayoshi Soma

**Affiliations:** 1 Department of Pathology, SASAKI Institute, Kyoundo Hospital, Chiyoda-ku, Tokyo, Japan; 2 Department of Cancer Genome Research, SASAKI Institute, Chiyoda-ku, Tokyo, Japan; 3 Department of Gynecology, SASAKI Institute, Kyoundo Hospital, Chiyoda-ku, Tokyo, Japan; 4 Department of Medical Oncology, SASAKI Institute, Kyoundo Hospital, Chiyoda-ku, Tokyo, Japan; 5 Department of Internal Medicine, SASAKI Institute, Kyoundo Hospital, Chiyoda-ku, Tokyo, Japan; University of Sharjah, UNITED ARAB EMIRATES

## Abstract

Formalin-fixed paraffin-embedded (FFPE) blocks are used as biomaterials for next-generation sequencing of cancer panels. Cross-contamination is detected in approximately 5% of the DNA extracted from FFPE samples, which reduces the detection rate of genetic abnormalities. There are no effective methods available for processing FFPE blocks that prevent cells from mixing with other specimens. The present study evaluated 897 sheets that could potentially prevent cell transmission but allow for the movement of various solvents used in FFPE blocks. According to the International Organization for Standardization and Japanese Industrial Standards, six requirements were established for the screening of packing sheets: 1) filter opening ≤5 μm, 2) thickness ≤100 μm, 3) chemical resistance, 4) permeability ≥1.0 × 10^−3^ cm/s, 5) water retention rate <200%, and 6) cell transit test (≤2 cells/10 high-power fields). Polyamide, polyethylene terephthalate, and polypropylene/polyethylene composite sheets met all criteria. A pocket, which was designed to wrap the tissue uniformly, was made of these sheets and was found to effectively block the entry of all cell types during FFPE block processing. Using a sheet pocket, no single cell from the cell pellet could pass through the outer layer. The presence or absence of the sheet pocket did not affect hematoxylin and eosin staining. When processing FFPE blocks as a biomaterial for next-generation sequencing, the sheet pocket was effective in preventing cross-contamination. This technology will in part support the precise translation of histopathological data into genome sequencing data in general pathology laboratories.

## Introduction

Formalin-fixed paraffin-embedded (FFPE) tissue blocks is a basic technology that supports pathology and clinical medicine. Its essential role is to prevent decay and supply reproducible staining specimens, such as H&E staining and immunostaining, for pathological diagnosis. Pathologists determine the diagnosis, which leads to the current standard treatment. Moreover, pathogenic nucleic acids derived from cancer cells exist in FFPE blocks, and the cancer is subsequently detected by pathologists.

FFPE blocks are the most important biomaterials used to determine somatic mutations in cancer samples using next-generation sequencing (NGS). NGS can detect genomic diversity, tumor heterogeneity, and small amounts of mitochondrial DNA and nuclear DNA [1–6]. To achieve high diagnostic precision, FFPE blocks should not allow contamination of the specimen. Although various methods or optimized workflows to prevent contamination of tissue fragments have been used over the last 100 years, conventional procedures for histopathological diagnosis based on morphology are insufficient to prevent contamination at the cellular level.

Sehn et al. found that nine of 296 (3%) clinical NGS cases showed cross-contamination of approximately 5% of DNA extracted from FFPE blocks, which was derived from other patients using a read haplotype-based approach [7]. Similar results were obtained from 230 cases using an NGS-based multiplex gene panel test, in which cross-contamination was detected in 3.9% of FFPE blocks using the ContEst program [8, 9]. In a report by the Japanese Ministry of Health, Labour and Welfare, 6 of 104 cases (5.8%) were detected with more than 1% contamination in the Trial of Onco-Panel for Gene-profiling to Estimate both Adverse Events and Response (TOP-GEAR) project. False-positive genetic abnormalities could be detected if the contaminated samples harbor clinically important somatic mutations that maintain the cancer state. Contamination may also lead to false-negative calls for somatic mutations that are present in the tested samples but not in the contaminated samples because the variant allele frequencies are reduced to levels below the detection threshold. Consequently, the reliability of the diagnosis is compromised, and patients are administered ineffective or harmful molecular-targeted drugs. Therefore, there is an urgent need to develop a tool to prevent cross-contamination at the cellular level during the preparation of FFPE specimens.

Various sheets, such as hydrophilic polyvinylidene fluoride (PVDF) and nitrocellulose membranes, have been used to collect biomaterials with sufficient quality and quantity of nucleic acids for genomic and regenerative medicine. The development of materials that retain cells while allowing solutions to pass through has been attempted in tissue engineering. For example, a scaffold material for constructing an artificial bronchus has been developed and its capacity for cell retention and water permeability has been investigated [10]. Dialysis membranes are another example of sheets used in medical practice for emergency use [11]. Antimicrobial filter membranes have also been developed by combining PVDF membranes, polyethersulfone membranes, and cellulose acetate with antimicrobial substances [12]. These sheets prevent the permeation of cells and/or microbes but allow various solutions to pass through; however, no sheet has been developed to prevent cross-contamination of FFPE blocks in NGS.

During the preparation of FFPE blocks, a sheet must be developed to block the permeation of cells from other samples/specimens. In this study, we screened sheets that prevented cell permeation and allowed various tissue-processing-related solutions to pass through. A pocket to wrap the cassette containing human tissue was designed from sheets screened for the required properties. The sheet pocket prevented cross-contamination during the FFPE process and showed great potential as a candidate biomaterial for NGS.

## Materials and methods

### Sheet selection

A total of 897 sheets for which samples and data were available were evaluated for the following six requirements: 1) average filter opening size ≤5 μm, 2) thickness ≤100 μm, 3) chemical resistance (JIS A 5209:2014, ISO 13006:2012), 4) permeability ≥1.0 × 10^−3^ cm/s (JIS1913:2019, ISO 9073:1995), 5) water retention rate <200% (JIS A1218:2009, ISO 11274:2019), and 6) cell transit test ≤2 cells/10 high-power fields (HPFs) (JIS K3835:1990, ISO 23033:2021). Each requirement was determined according to the Japanese Industrial Standard (JIS) and International Organization for Standardization (ISO). The protocols for the screening procedure are described in the S1 File and published on protocols.io, https://dx.doi.org/10.17504/protocols.io.b2fyqbpw. The evaluation was conducted in a pathology laboratory that complied with JIS8703:1983 and ISO554:1976 (temperature, 23°C; relative humidity, 50%).

### Evaluation of sheet pockets in FFPE tissue models

**Fig 1:**
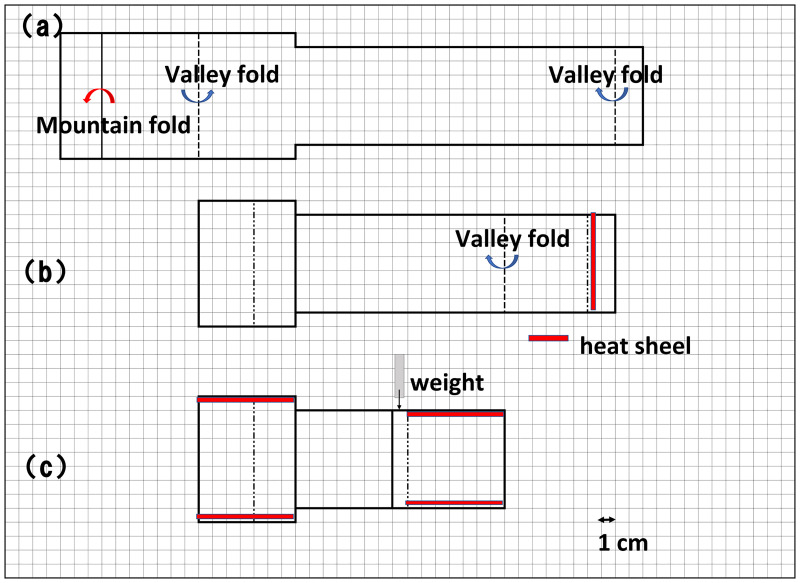
The development of a sheet pocket. Illustration of the three folding steps for pocket creation. (A) Mountain folding (the fold is made by folding the bottom edge behind the sheet and away from you) along the solid line and valley folding (the fold is made by folding the bottom edge of the sheet up towards you and then down to lay over the surface of the sheet) along the dashed line. (B) The addition of a heat seal to create the weight insertion area. A weight was inserted when the pocket was not fully immersed in the solution because of an air pocket. (C) Adhesion with a heat seal to create two pockets.

To evaluate the blocking effects, sponge pieces were used as tissue models instead of human tissues. The pocket was designed to wrap the sponge-containing cassette properly and securely (Fig 1). The sheet pocket has two compartments at either end of the sheet. The cassette was placed in one compartment, the pocket was folded three times, and then the cassette was placed into the second compartment and sealed using a closing lip. The wrapping procedure is shown in the S1 Video.

Unwrapped and wrapped cassettes were immersed in a 10% buffered formalin solution containing a high concentration of cells (1.0 × 10^7^ cells/mL) present in the pleural fluid and mixed at 23°C overnight. After removal from the mixture, the FFPE blocks were processed according to the standard operating procedure. Three 4 μm-thick slices were cut from the specimens and hematoxylin and eosin (H&E) staining was performed on glass slides. The number of nucleated cells within the sponge was counted in 10 consecutive fields of ×400 HPF under a microscope. The average number of cells in the sponge was calculated as the sum of these counts. The above tests were performed three times using a triple-layered pocket made of the PP/PE composite synthetic material to reproduce the experiment.

### Effect of a sheet pocket of cell block

The cell block was a special FFPE block made from a cell pellet. Floating single or clustered cells in the pleural fluid were centrifuged at 1,500 × 100 mm for 5 min and fixed with 10% formalin buffer overnight. The cell pellet was placed in the cassette, together with the cut end of the centrifuge tube, and wrapped in a triple-layered pocket made of PP/PE composite synthetic material. After paraffin impregnation, the inner side of the sheet was sampled to obtain H&E-stained specimens, and the surface conditions of the sheets were observed under a microscope. To examine the presence or absence of cells passing through the packing sheet from the cell block, the paraffin used for treatment was processed into a paraffin block, and five deeper-cut sections were observed under a microscope.

### Evaluation of H&E staining of tissue specimens

The differences in H&E staining between blocks made by wrapping a triple-layered pocket made of PP/PE composite synthetic material and normal blocks were compared in 100 cancer tissue sections. H&E staining was performed according to the standard procedure using an external control slide. The stained specimens were independently reviewed by one pathologist (KI) and three pathology laboratory technicians (NT, YM, and RO) and compared in terms of density, sharpness, clarity, and contrast of H&E staining.

This study was approved by the Institutional Review Board (IRB) of the SASAKI Institute. Written informed consent was obtained from all patients. All medical records were fully anonymized in the Clinical Biobank of the SASAKI Institute.

### Statistical analysis

The number of cells in both the cell transit tests and FFPE tissue models was evaluated using Student’s t-test. Statistical analysis was performed using one-way analysis of variance (ANOVA), followed by a post-hoc multiple comparison test (Tukey’s test). These analyses were performed using the Medical and Pharmaceutical Statistics (MEPHAS) (https://alain003.phs.osaka-u.ac.jp/mephas/). Statistical significance was set at *P* < 0.05.

## Results

### Sheet selection

**Fig 2:**
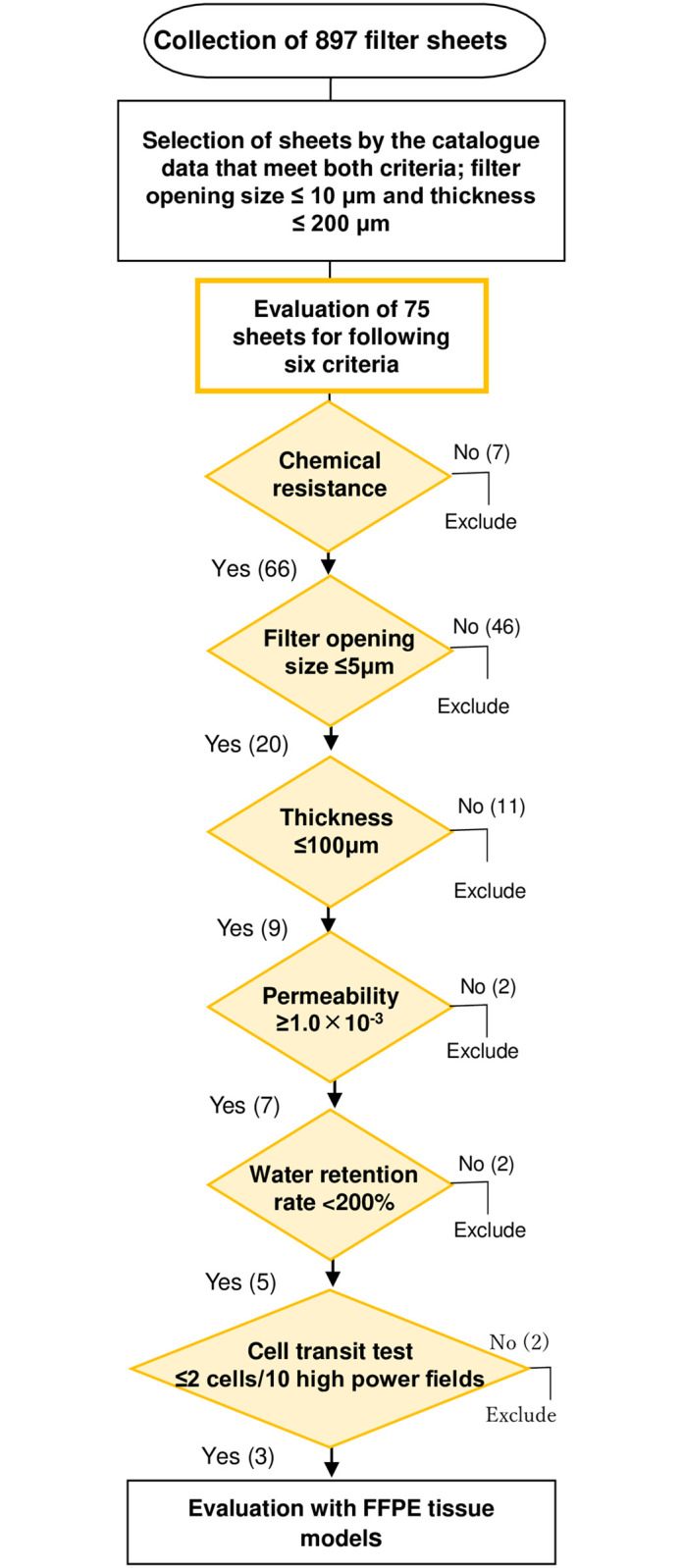
Workflow of sheet selection. Summary of selection strategy and criteria for sub-selection.

A total of 897 sheets were screened according to a standard workflow (Fig 2), which first considered filter openings ≤10 μm and thicknesses ≤200 μm. We obtained 75 sheets from several companies that met these criteria and continued the evaluation according to the six optimal criteria. Xylene is an organic solvent used to prepare FFPE blocks in pathology laboratories. Seven of the 75 sheets were disqualified owing to their incompatibility with xylene, including nitrocellulose and polyvinylidene fluoride membranes. Xylene-resistant sheets are made of materials such as polyamide (PA, Nylon^Ⓡ^), polyethylene terephthalate (PET), polypropylene (PP)/polyethylene (PE) composites, PET/PA composites, and hydrophilic polytetrafluoroethylene (PTFE-aq), which are also insoluble in formalin, alcohol, and paraffin. Filter opening size is an important structural factor that determines the sheet permeability of different cell types. Accordingly, 46 sheets with pore sizes of > 5 μm were excluded. Sheets with multi-laminated structures are thick and do not allow cells to permeate. However, their thickness can render them incompatible with certain devices used in pathology laboratories. Notably, a water retention rate higher than 200% causes sheets to expand during the preparation of the FFPE blocks, causing the same problems as in thick sheets.

The water permeability test was conducted on nine sheets made from five xylene-resistant materials with filter openings of <5 μm (Table 1). Of these, the PTFE-aq sheet and one PET sheet did not meet the permeability criteria, and were likely to interfere with the rapid solvent displacement during the preparation of FFPE blocks. In the water retention test (Table 2), the two PET/PA sheets did not satisfy the minimum criterion (<200%). Three sheets that met all six criteria were selected (Table 3).

**Table 1:** Water permeability test.

Company	A		B	C			D		E
Filter sheets (materials)	PA	PET	PET	PP/PE			PET/PA		PTFE-aq
**Filter opening (μm)**	1	1	1	5	5	2	1	2	1
**Thickness (μm)**	75	65	70	85	20	75	630	410	50
**measurement time(s)**	49.99	170.48	36.11	5.84	11.52	29.52	75.79	201.47	665.66
	49.01	169.55	37.38	5.64	11.2	29.65	76.11	202.24	684.99
	47.77	171.15	38.42	5.78	11.76	29.35	76.86	206.63	706.68
	49.11	170.12	36.67	5.9	11.65	29.08	76.32	202.36	734.33
	49.49	166.88	37.22	5.95	11.62	30.09	76.18	203.99	778.75
**average**	49.07	169.64	37.16	5.82	11.55	29.54	76.25	203.34	714.08
**permeability κ(cm/s)**	3.2x10^-3^	7.8x10^-4^	4.0x10^-3^	3.1x10^-2^	3.7x10^-3^	6.5x10^-3^	2.8x10^-2^	1.0x10^-2^	2.5x10^-5^

PA: Polyamide 66

PET: polyethylene terephthalate

PP/PE: polypropylene/polyethylene

PTFE-aq: hydrophilic polytetrafluoroethylene

**Table 2:** Water retention test.

Company	A		B	C			D	
Filter sheets (materials)	PA	PET	PET	PP/PE			PET/PA	
**water retenion rate (%)**	38.1	39.5	58.3	208.5	51.3	113.3	515.9	344.2
	18.4	22.5	45.3	128.2	48.8	105.7	531.2	347.7
	45.1	34.2	65.4	144.0	56.3	107.3	539.4	334.5
**average**	33.9	32.1	56.3	160.2	52.1	112.1	528.9	342.1

PA: Polyamide 66

PET: polyethylene terephthalate

PP/PE: polypropylene/polyethylene

PTFE-aq: hydrophilic polytetrafluoroethylene

**Table 3:** Three sheets met the six criteria.

Materials	Filter opening (μm)	Thickness (μm)	Chemical resistance	Permeability κ(cm/s)	Water retention rate (%)	Cell transit test (number/10 HPFs†)
**PA**	1	75	〇	3.2x10^-3^	33.9	2≧
**PET**	1	70	〇	4.0x10^-3^	56.3	2≧
**PP/PE**	2	75	〇	6.5x10^-3^	112.1	2≧

PA: Polyamide 66

PET: polyethylene terephthalate

PP/PE: polypropylene/polyethylene

^†^HPF: high power field

**Fig 3:**
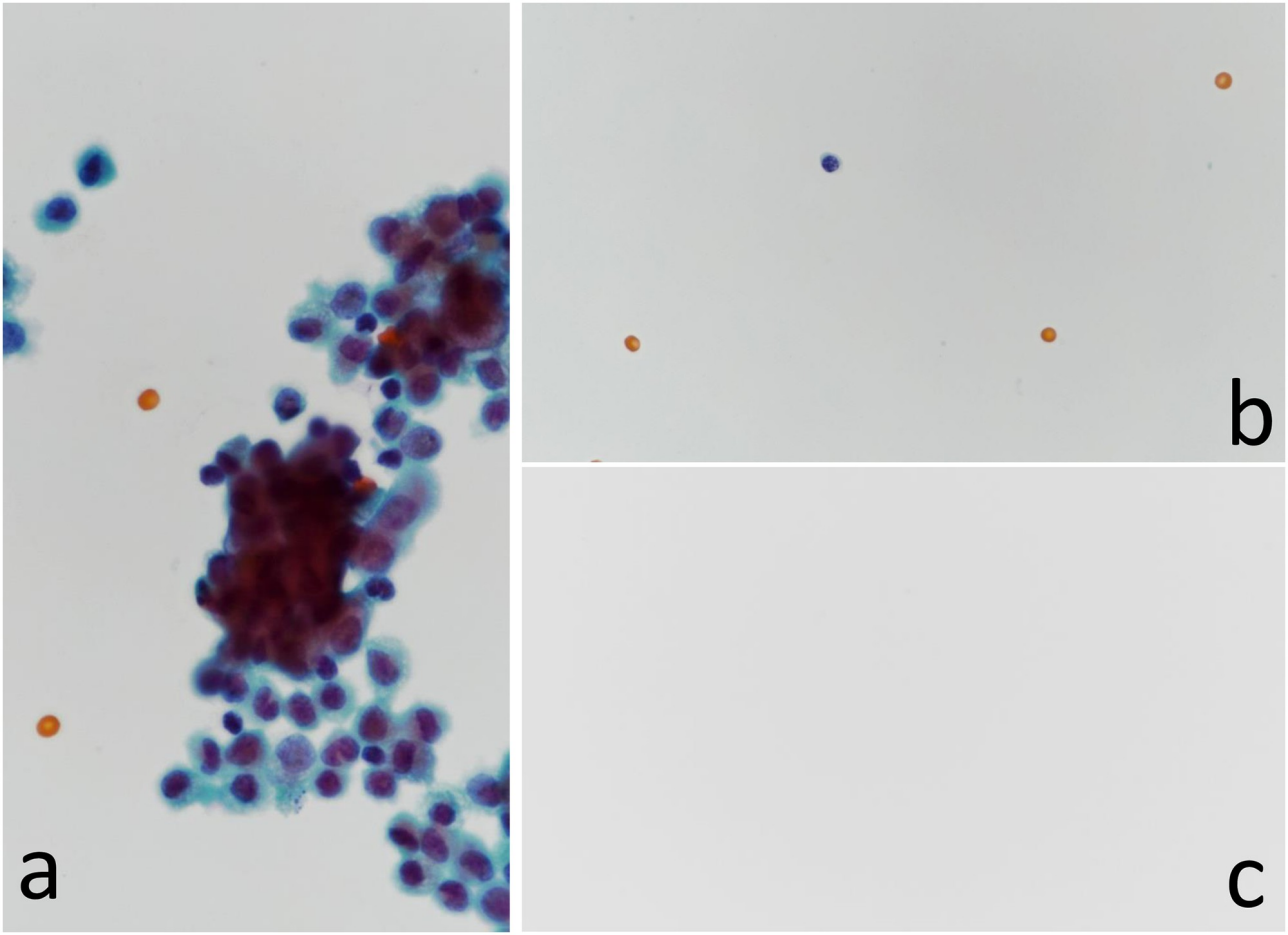
Microscopic findings of cell transit test. (A) Dense fluid (1.0 × 10^6^ cells/mL) stained with Papanicolaou (original magnification ×400). Mesothelial cells, various inflammatory cells, and erythrocytes can be seen. (B) Filtrate with a single-layered sheet (original magnification ×400). One nucleated cell is detected with three erythrocytes. (C) Filtrate with three-layered sheets (original magnification ×400). A complete absence of cells and erythrocytes can be seen.

However, because the nucleated cells could not be completely blocked and contained erythrocytes, we evaluated the efficacy of the double- and triple-layered PP/PE composite synthetic sheets. The number of erythrocytes penetrating through the sheets was found to be significantly lower in triple-layered than in double-layered (*P* < 0.05) and single-layered sheets (*P* < 0.05; Table 4). A significant reduction in the number of permeating nucleated cells was observed in the triple-layered sheets compared with the single-layered sheets (*P* < 0.05); however, there was no difference in permeability between the single- and double-layered sheets. Fig 3C shows that any type of cell passed through the triple-layered sheets; however, a few nucleated cells and erythrocytes permeated through the single-layered sheets (Fig 3B).

**Table 4:** Cell transit test according to the number of layers.

	Sheet (+)			Sheet(-)
	1 layer	2 layers	3 layers	
**number of nucleated cells**	2	0	0	12.5x10^3^
	3	0	0	10.7x10^3^
	1	2	0	9.6x10^3^
**average**	2	0.67	0	10.9x10^3^
**number of erythrocytes**	118	89	0	3.6x10^3^
	118	91	0	4.2x10^3^
	139	76	0	5.6x10^3^
**average**	125	85.3	0	4.5x10^3^

### Evaluation of sheet pockets in FFPE tissue models

**Fig 4:**
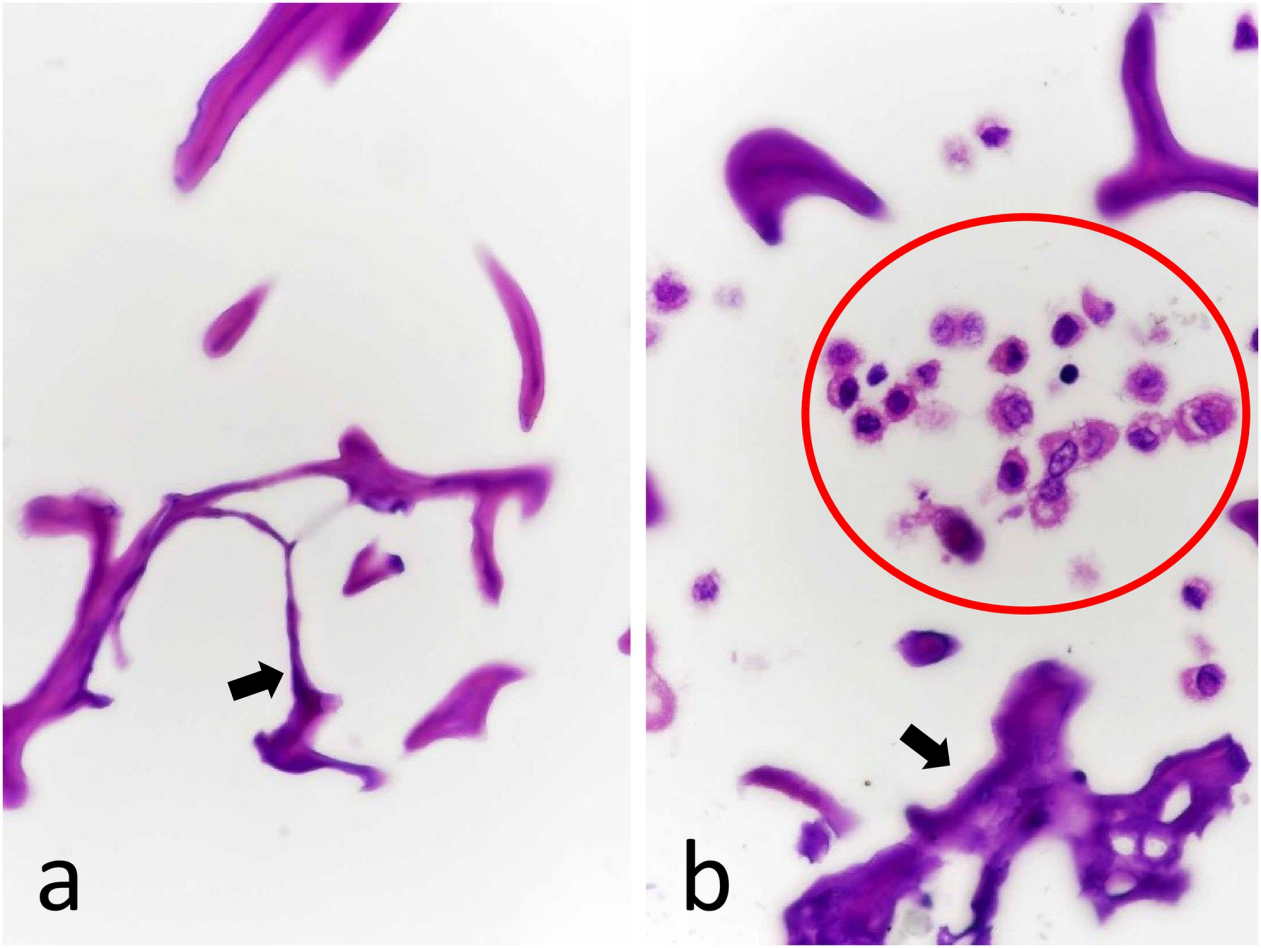
FFPE tissue model using sponge pieces. To evaluate the blocking effects from contamination, a sponge piece was used as a tissue model instead of a human tissue section. (A) H&E-stained specimen of FFPE model using a sheet pocket. No nucleated cells were observed (original magnification, ×400). The black arrow indicates sponge. (B) H&E-stained specimen of the FFPE model without a sheet pocket. Many nucleated cells (red circle) were detected within the air space of the sponge (original magnification, ×400).

Three-layered pockets with one compartment each were made of PA, PET, and PP/PE composite synthetic sheets. Sponge-containing cassettes were inserted into these pockets, which prevented 99.9% of all cell types from entering the cassette. There were no significant differences in the number of cells entering the sponges between the pockets made of PA, PET, and PP/PE composite synthetic sheets (Table 5). A triple-layered pocket made of a PP/PE composite synthetic material was used to reproduce the experiment; however, several nucleated cells were observed as clusters within the sponges. Considering the filter opening size, we assumed that the cells observed in the PA, PET, and PP/PE composite synthetic sheets may have entered through the entrance rather than penetrating the three layers. Therefore, we evaluated the efficacy of a design in which the entrance of the pocket was either bound by a wire or folded and enclosed in a second compartment (i.e., the compartment entrance was folded into the pocket). No obvious reduction in the number of infiltrating cells was observed when the entrance to the pockets was tied with wires; however, a significant decrease was detected when the cassette compartment entrance was folded into the pocket (*P* < 0.05) (Table 6). The pocket designed with the two compartments showed a 100% prevention rate (Fig 4).

**Table 5:** Wrapping effects according to materials in FFPE tissue models.

materials	pocket (+)			pocket (-)
	PA	PET	PP/PE	
**number of cells (nucleated cells & erythrocytes)**	1	2	0	50.5x10^2^
	0	0	0	28.6x10^2^
	0	0	5	51.1x10^2^
	0	0	0	29.5x10^2^
	1	0	0	32.7x10^2^
**average**	0.4	0.4	1	38.5x10^2^

PA: Polyamide 66

PET: polyethylene terephthalate

PP/PE: polypropylene/polyethylene

**Table 6:** Wrapping effects according to pocket compartment design in FFPE tissue models.

	pocket (+)			pocket (-)
	one compartment	one with wire	two compartment	
**number of cells (nucleated cells & erythrocytes)**	5	1	0	26.4x10^2^
	3	0	0	27.2x10^2^
	6	0	0	52.9x10^2^
	1	5	0	51.7x10^2^
	5	1	0	36.2x10^2^
**average**	4	1.4	0	38.9x10^2^

### Effect of a sheet pocket of cell block

**Fig 5:**
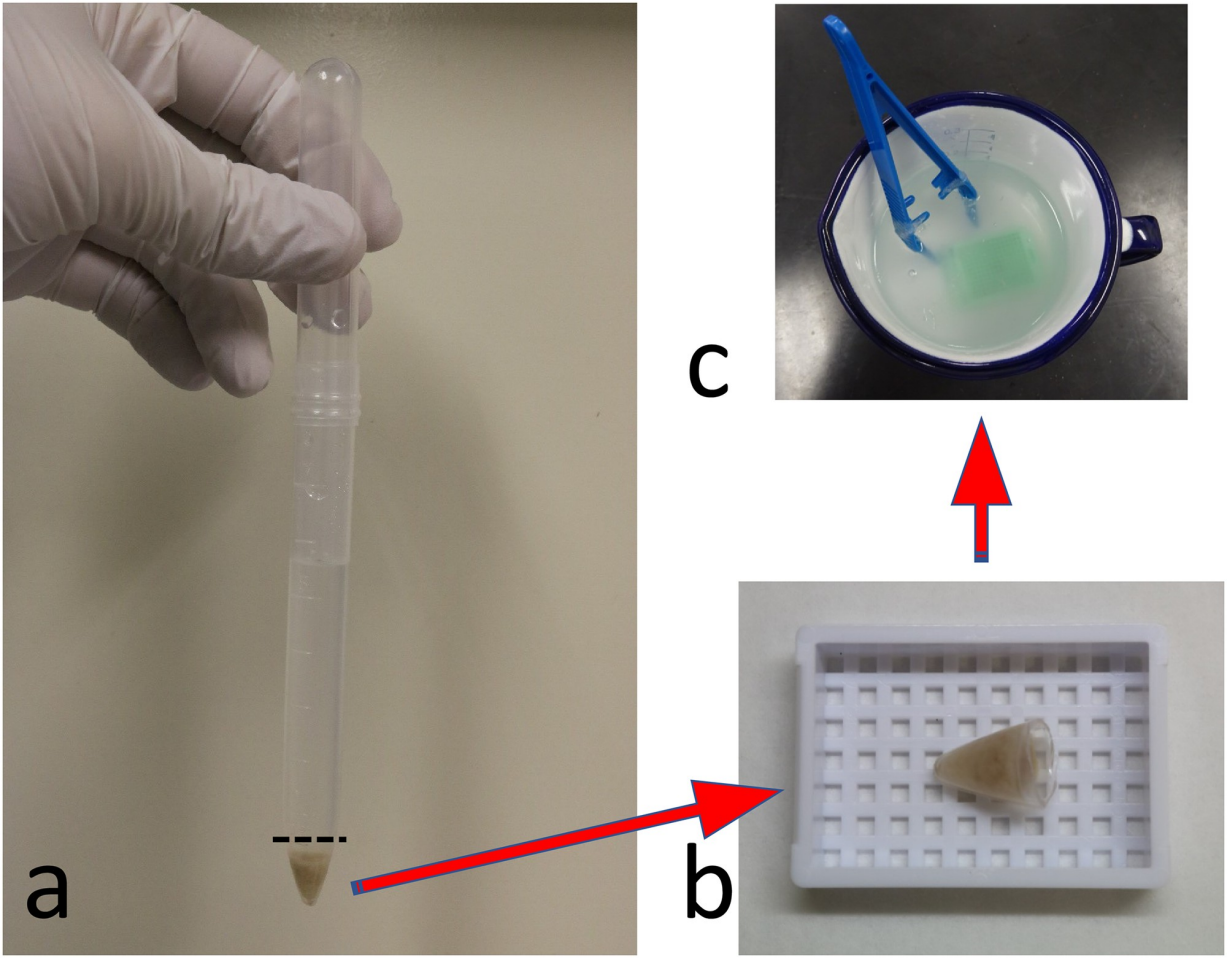
Procedure of cell block. (A) Cell pellet made from pleural fluid. Floating single or clustered cells in the pleural fluid were centrifuged at 1,500 × 100 *g* for 5 min. (B) Cell pellet in the cassette. The cell pellet was placed in the cassette together with the cut end of the centrifuge tube. (C) Paraffin embedding within each cup.

**Fig 6:**
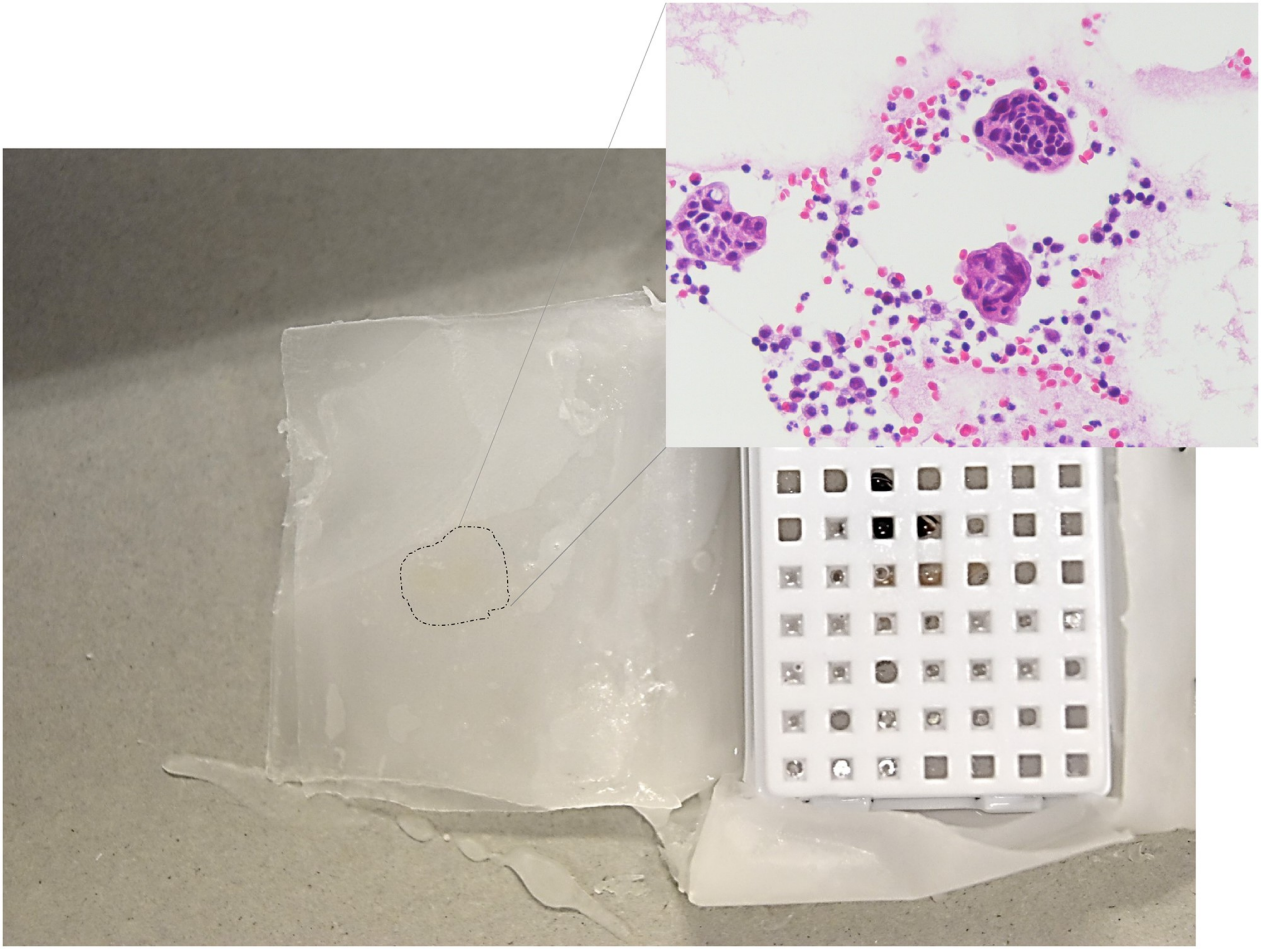
Detection of cells dissociated from cell pellet placed in the cassette. After paraffin impregnation, a slightly yellow smudge was detected on the sheet. A section of the sheet was prepared in this area for H&E staining. Cancer and inflammatory cells were observed on the surface of the sheets.

**Fig 7:**
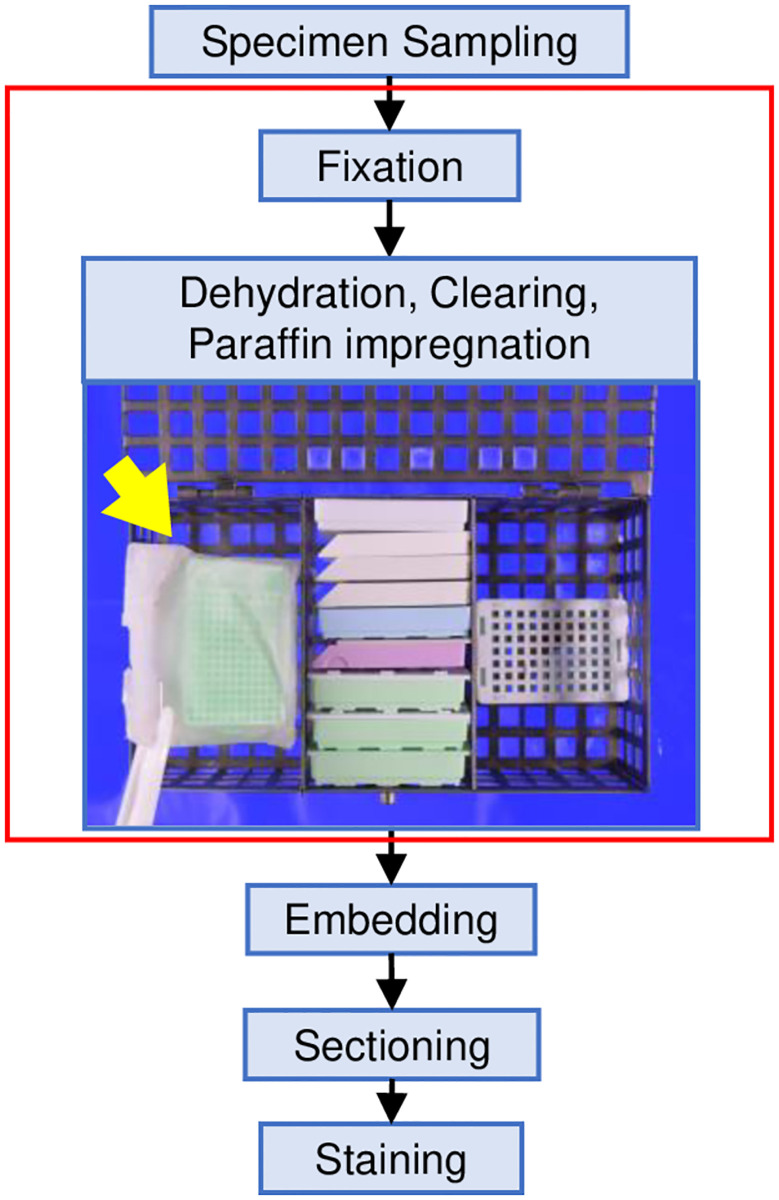
A block diagram of the key steps of the FFPE procedure. Wrapping of the cell pellet in the cassette prevented contamination during FFPE processing, as indicated by the red line in the block diagram. The sheet pocket is indicated by the yellow arrow.

The cell block procedure is shown in Fig 5. Paraffin embedding was performed for each cup. When the inside of the sheet opened after paraffin impregnation was examined under a microscope, cells that leaked from the cassette were found to adhere to the inside of the sheet (Fig 6). In the examination of the paraffin used in impregnation, no obvious passage of cells outside the sheet was observed. This procedure was repeated three times to obtain the same results. Wrapping the cells with a double-compartment-designed sheet made of the PP/PE composite synthetic material prevented cross-contamination that arose during FFPE block processing (Fig 7).

### Evaluation of H&E staining of tissue specimens

**Fig 8:**
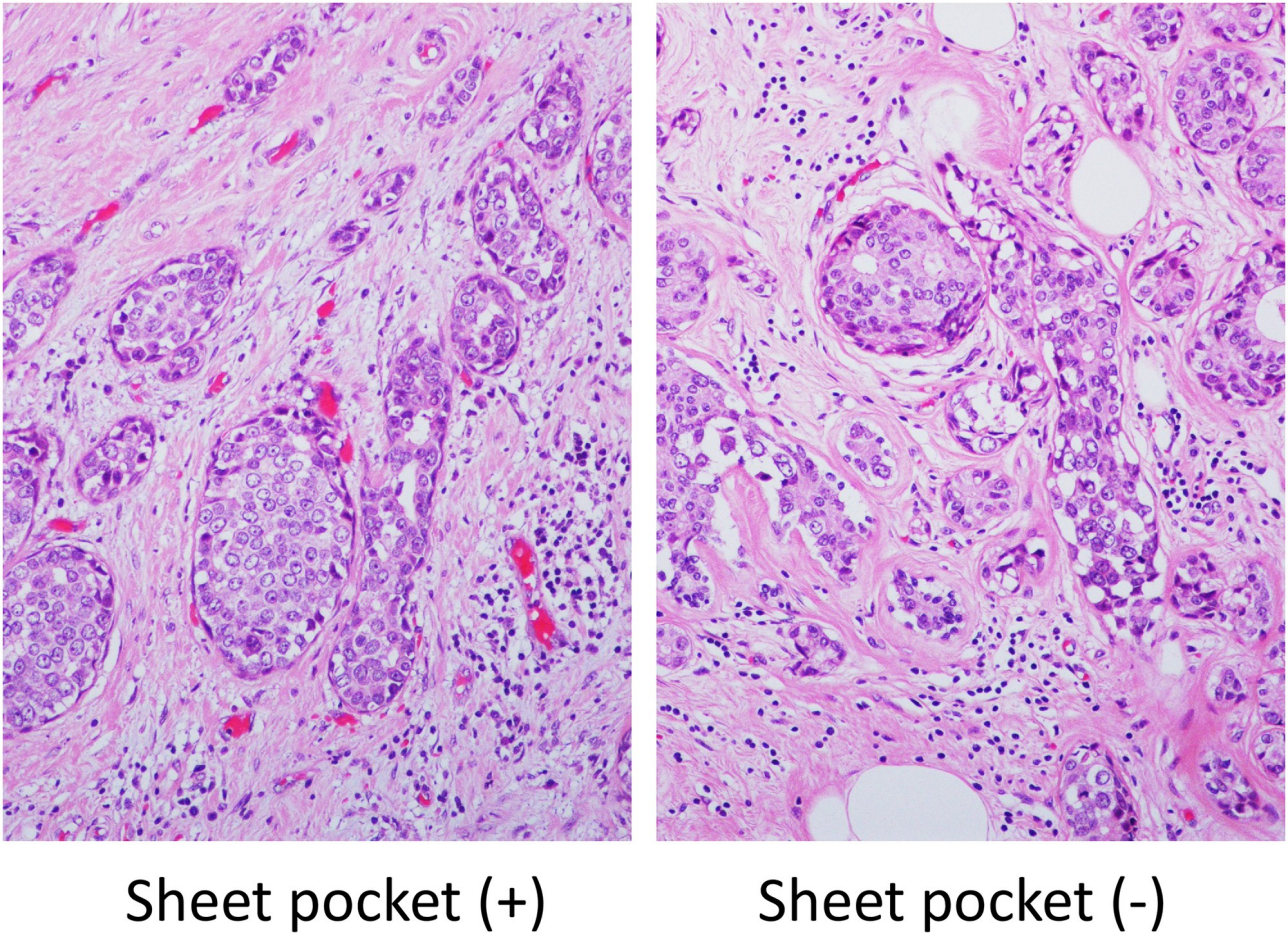
Influence of staining properties of H&E staining tissue specimens. No differences in H&E staining were detected between the blocks using a sheet pocket and those using normal blocks.

The difference in H&E staining between the blocks made using a sheet pocket and normal blocks was examined. Various tumor tissue sections were used for comparison. No differences in H&E staining were observed between specimens (Fig 8). In addition, the use of the wrapping material did not interfere with the elongation and debridement of the tissue specimens. There was no difference in H&E staining between specimens with and without sheet pocket wrapping. No differences in H&E staining were detected between the blocks using a sheet pocket and those using normal blocks.

## Discussion

Modern genetic information has shifted from pathological diagnoses to therapeutic targets in medicine [13]. FFPE blocks, which reliably contains pathogenic genes, is the most frequently used biomaterial in clinical genetic examination. However, they require adjustments for improved functionality, including higher maintenance of nucleic acid quality and prevention of contamination from other specimens during processing.

With the establishment and publication of regulations for FFPE tissue specimen handling for genome research and medical treatment, the maintenance of nucleic acid quality in FFPE blocks has been addressed [14–18]. However, specimen contamination during routine clinical NGS is yet to be determined.

By using packaging sheets, we aimed to prevent cellular cross-contamination, which typically occurs in a pathology laboratory. This study showed that the ideal sheet should meet at least six specific structural criteria, and an effective pocket design should include two compartments at either end of the sheet. The tissue-containing cassette was placed in the small compartment, folded thrice, inserted into the large compartment, and sealed using a closing lip. The sheets designed in this study prevented passage of all cell types, including erythrocytes. Erythrocytes do not have nuclei and thus lack nucleic acids; however, mature erythrocytes express high levels of circular RNA [19]. Because erythrocytes exist in all tissues, contamination of erythrocytes from other samples could adversely affect precision medicine.

Cell block is a special FFPE blocking technique that collects floating cells in ascites and pleural fluid. The present study showed that cells leaked from the cassette, and that the use of a sheet pocket prevented cell passage. Therefore, the use of the sheet pocket is an effective method for preventing contamination at the cellular level in a pathology laboratory.

FFPE blocks do not require deep freezers or liquid-nitrogen tanks for banking. This greatly reduces the biohazard risk and allows easy transport at room temperature. FFPE blocks, which complies with the accuracy control standards for genome research, will contribute to genome drug discovery as a biomaterial for clinical biobanks. This study aimed to develop a technical basis for adapting FFPE blocks to NGS, while reducing procedural expenses.

To prevent preanalytical cross-contamination in genetic testing, we propose the use of wrapping sheets that effectively prevent cellular passage. This study lacked validation data on the cross-contamination contained in FFPE blocks. Clinically, the number of NGS-based multiplex cancer panel tests is rapidly increasing, and validation data for FFPE blocks are essential for future studies. Prospective studies that evaluate cross-contamination using precise *in silico* procedures will promote the development of highly accurate genetic testing. It is necessary to construct a new testing platform where pathology and genetic testing laboratories can work together.

## Conclusions

To prevent the cross-contamination of FFPE blocks as a biomaterial for NGS, a sheet pocket was created and proposed for use in routine pathology diagnostics. This study demonstrated that a sheet pocket can block the permeation of single cells and prevent cross-contamination. A sheet pocket was designed using a folding procedure that can easily be used by any clinical laboratory technician.

## Supporting information

S1 File Detailed operation manual of six requirements. (PDF)

S1 Video Simple rapping procedure: How to use a sheet pocket. The cassette was inserted into a small compartment. The compartment was folded thrice and inserted into a larger compartment. Finally, the closed lip seals off the larger compartment.
(MP4)
