# Supplementary material for: A sheet pocket to prevent cross-contamination of formalin-fixed paraffin-embedded block for application in next generation sequencing
Source: PLoS One. 2022 May 4;17(5):e0266947. doi: 10.1371/journal.pone.0266947 (PMC9067696; doi:10.1371/journal.pone.0266947)
Supplement: S1 File — (PDF) [file pone.0266947.s001.pdf]

Protocol for micro-sheet pocket 1: Detailed operation manual of six requirements

iwaya
iwaya : iwaya@po.kyoundo.jp

1

iwaya

This protocol details micro-sheet pocket 1 and detailed operation manual of six requirements.

f2bbbpbgf.pdf

iwaya . Protocol for micro-sheet pocket 1: Detailed operation manual of six requirements. **protocols.io**
https://protocols.io/view/protocol-for-micro-sheet-pocket-1-detailed-operati-b2fyqbpw

micro-sheet pocket , permeability test device

Nov 30, 2021

Dec 02, 2021

Nov 30, 2021 maria.s

Dec 02, 2021 iwaya

55512

- Test environment: A horizontal worktable in a laboratory in accordance with JIS 8703-1983, ISO 554-1976 (temperature 23°C, relative humidity 50%)

Water permeability test:

Equipment / Consumables

- 1 x **50 mL** plastic measuring pipette
- P1000 Pipette Tips (without filter) (Gilson) 1 pc
- P200 Pipette Tips (without filter) (Gilson) 2 pcs
- Container 1 (at least 8-10 cm in diameter and 15 cm deep, made of a material that 34 can be easily pierced) for example, 1 L-PET bottle.
- Container 2 (at least 15 cm in diameter and at least 5 cm in depth)

- Burette stand
- Parafilm
- stopwatch (measures up to 2 decimal places)
- funnel
- **500 mL** beaker
- Scissors and/or cutter
- plastic cutter
- drill

Solution :

500 mL distilled water

Test sheets (cut with scissors and/or cutter to 3cm x 3cm square).

Cell transit test:

Equipment / Consumables

- P1000 Pipette Tips (without filter) (Gilson) 1 pc
- P100 Pipette (Gilson) 2 pcs
- P100 Pipette
- P20 Pipette
- P20 Pipette Tips
- **2 mL** micro tube
- **1.5 mL** / **2 mL** microtube rack
- microscope slide
- alcohol-resistant marking pen
- optical microscope (400x)
- aspirator
- **50 mL** plastic centrifuge tube
- cell counterplate (Watson, Neubauer Improved)
- Parafilm
- Scissors

Solution/Reagent:

- 10% neutral buffered formalin solution
- Residual specimen (ascites or pleural effusion) or cultured cells (1 x 10^6-10^7 cells)
- PBS 70mL
- 0.4% Trypan blue solution
- 95% Ethanol
- Papanicolaou staining solution

Average diameter of filter opening

1 Examine the average diameter of filter opening by referring to the catalog of the sheet.

2 Observe under a polarized light microscope for sheets with a diameter of filter opening of less than **10 µm** .

3 Confirm opening size by using scanning electron microscopy (SEM), if possible.

Thickness

4 Examine the thickness by referring to the catalog of the sheet.

5 Prepare 10 test sheets (cut with scissors to **20 cm** x **20 cm** square).

6 Put 10 test sheets on a horizontal worktable.

7 Lay a glass plate (200 ± 2 mm square, a mass of 82 ± 2 g, a thickness of **0.7 mm**) on the test sheets.

8 After about **00:00:10** , measure the test pieces to **0.1 mm** using a vertical ruler. 10s

Chemical resistance

9 Cut in half the sheet in order to compare each other.

10 Immerse the whole part of one cut sheet in the test solution (xylene).

11 Cover the sheet with a lid and kept at Room temperature for about 08:00:00 . 8h

12

Wash with water, dried, and observe both samples.

13 If no obvious change is observed, repeat the procedure 9-12 by changing the test solution (formalin, methanol).

Water permeability test

14 **Make-up for water permeability test device**

14.1 Create a water storage tank

Cut off the suction opening of the 50 mL disposable measuring pipette with a plastic cutter.

14.2 Create drainage tank 1

Drill a hole to make an overflow port at a height of about 12 cm from the bottom of the container 1 (inner diameter of hole, about 15 mm) .

15 **Create a sample reservoir**

15.1 Cut the attachment part (a) of the tip of the P1000 pipette with scissors from the tip body (b) indicated by red arrow.

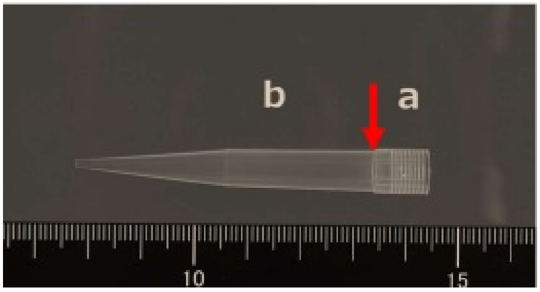

15.2 For one sheet, cut off at 2 cm from the tip of (b). In the case of two or more sheets, cut off 1.5 cm from the tip. (Red line).

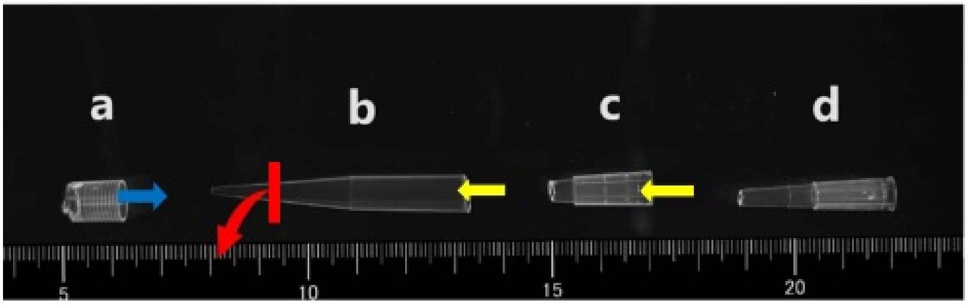

15.3 Measure the inner diameter (cm) of the cut side of (b) and calculate the cross-sectional area (inner diameter x inner diameter x 3.14) (Cross-sectional area of test sheet).

15.4 Cut a 2 cm area from the opening of the tip of the P200 pipette with scissors (c).

15.5 Cut a 3 cm area from the opening of the other P200 pipette tip with scissors (d).

15.6 Connect (b) Connect (c) and (d) (yellow arrow).

15.7 Cover (b) with the test sheet (3cm×3cm) on the red line.

15.8 Fix the sheet by fitting (a) (blue arrow).

15.9 Cut the sheet that protrudes from (a).

15.10 Seal and fix the connected parts with Parafilm.

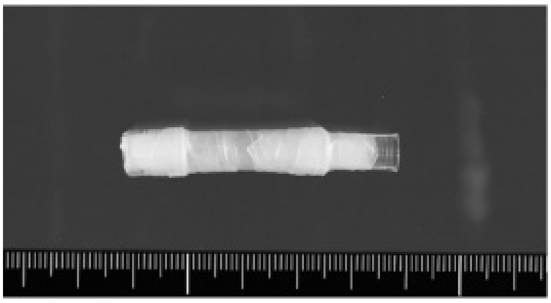

Complete view of the sample reservoir

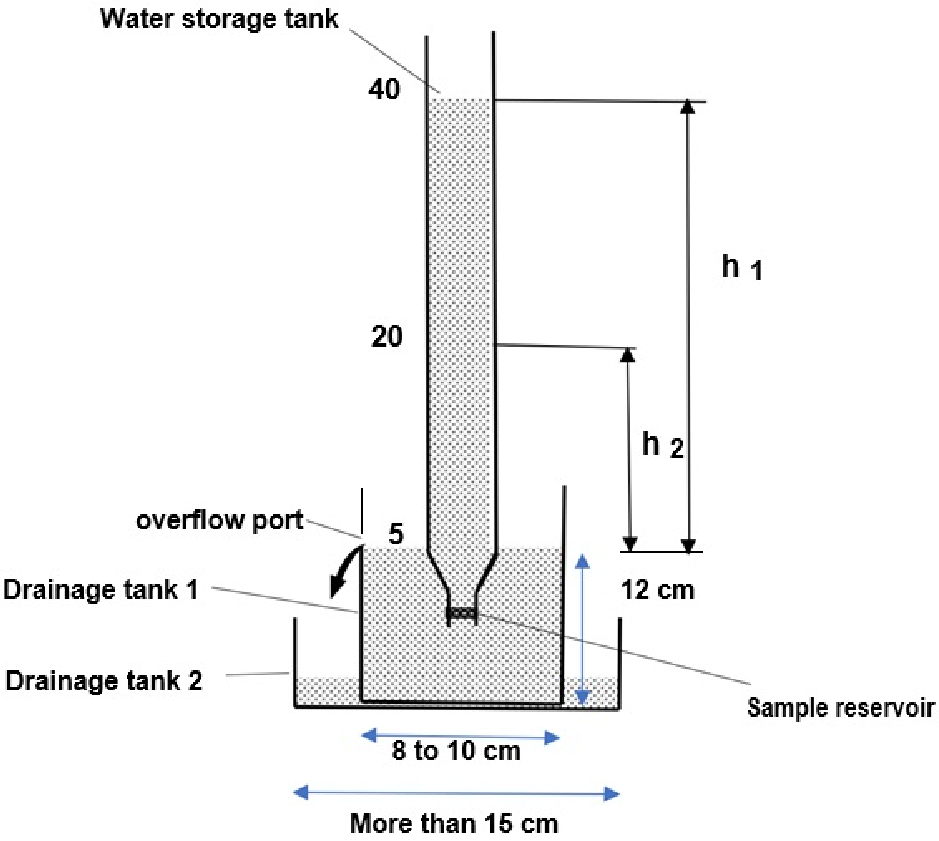

Assembly diagram of permeability test device

16 Vertical installation of a water storage tank on the workbench using a Burette stand.

17 Assemble water storage tank, drainage tank 1, drainage tank 2, according to the reference above diagram.

18 Adjust the height of the overflow opening to " 5 mL " on the scale of a 50 mL disposable measuring pipette.

19 Attach the sample reservoir to the end of the reservoir.

20 Wrap the parafilm to prevent liquid leakage.

21 Operation Procedure

21.1 Fill drainage tank 1 with water until it flows out of the overflow port.

21.2 Fill water storage tank with water by using a funnel.

21.3 Measure transit time (t2-t1 value) it takes for the water in a water storage tank to pass from the " 40 mL " scale to the " 20 mL " scale using a stopwatch.

21.4 Repeat the operation in steps 21.1 and 21.2 and confirm that the t2-t1 value was almost constant.

21.5 Measure t2-t1 value 5 times.

22 Calculation

The permeability coefficient was calculated by the following formula, applying JIS A 1218:2020.

$$k = 2.303\frac{aL}{A(t_2 - t_1)}\log_{10}\frac{h_1}{h_2}$$

κ :		permeability coefficient	cm/s		
a:		cross-section area of storage tank	cm2		
L:		thickness of test sheet	cm		
A:		cross-section area of test sheet	cm2		
t_2-t_1:		measurement time	s		
h_1:		water level difference at time t1	cm		
h_2:		water level difference at time t2	cm		

Water retention test

23 Prepare 3 sheets cut in 100 mm x 100 mm sized square.

24 Measure the mass of a cut sheet in the standard condition to **1 mg** -level.

25 Immerse 3 sheets in the water for at least **00:15:00** . 15m

26 Remove 3 sheets from the water with tweezers.

27 Allow the water to drip off for at least **00:01:00** 1m

28 Measure the mass of a cut sheet after wetting and dripping off the water (m2).

29 Calculate the water retention rate by the following formula

m=(m2-m1)/m1 x 100

m: water retention rate (%)

m1: mass of the specimen in the standard condition (mg)

m2: mass of the specimen after wetting and dripping off the water (mg)

30 Calculate the average value in accordance with JIS A 5209:2014, Rule B (rounding method).

Cell transit test 20m 5s

31 **Preparation of sample reservoir**

Preparation of sample reservoir (consisting of part (a) and (b), we named“filter assembly”) is explained in step 15.1

32 Set sample reservoir onto **2 mL** microtube.

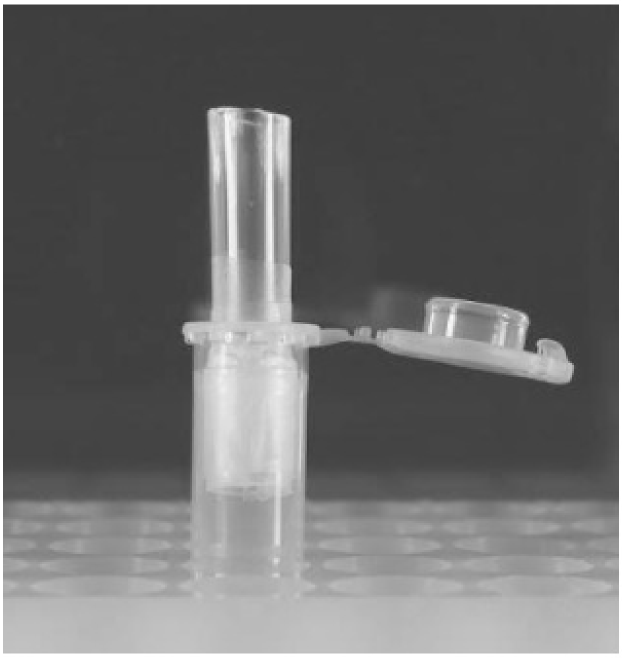

33 **Preparation of cell suspension (collection of cells from ascites and/or pleural fluid)**

33.1 5m

Centrifugation residual specimens (ascites and/or pleural effusion)at

2000 rpm for 00:05:00 .

33.2 Gently remove the supernatant with an aspirator.

33.3 Collect more than 1 mL of cell pellet.

33.4

Add 30 mL to 35 mL of PBS and mix by inverting.

33.5 Stand still for 00:15:00 . 15m

33.6 Gently remove the supernatant with an aspirator.

33.7

Repeat PBS wash (step 32.4 - 32.6).

33.8 5s

Counts all cells in a cell suspension

1. Add 90 µl of 0.4% trypan blue solution to 10 µl of cell suspension (10-fold dilution).
2. Gentle vortex for 00:00:02 - 00:00:03 .
3. Inject 6 µl samples with a P20 pipette through the sample inlet of the cell counter plate.
4. Count the number of cells in 4 compartments using the cell counter plate.
5. Calculate the number of cells per 1 µl of stock solution {(average number of cells of 4 compartments) × 10 × (dilution factor)}.

Note : The count of one plot should be about 100.

If the number of cells is large, dilute the cell suspension 10 to 100 times with PBS. If the number of cells is small, reduce the amount of 0.4% trypan blue solution added to the cell suspension 10 µl .

34 Adjust to 1 x 10^{6}- 10^{7} cells/ml in 10% neutral buffered formalin solution.

35 **Operation Procedure**

35.1

Apply **600 µl** of cell suspension (1 x 10^{6}-10^{7} cells/ml) to the sample reservoir (filter assembly) with a P1000 pipette.

35.2 Allow to natural filtration.

35.3

Measure the number of cells in the filtrate

1. Write an enclosure on a glass slide with an alcohol-resistant marking pen.
2. Add **15 µl** drops of 95% ethanol in the enclosure, followed promptly by **15 µl** drops of filtrate.
3. Allow to natural drying.
4. Perform Papanicolaou staining.
5. Count cells in 10 fields of view at 400x under the microscopy.
